# The role of estrogen receptor beta in breast cancer

**DOI:** 10.1186/s40364-020-00223-2

**Published:** 2020-09-07

**Authors:** Yujing Zhou, Xingdang Liu

**Affiliations:** Department of Nuclear Medicine, Huashan Hospital, Fudan University, No.12 Urumchi Middle Road, Jing’an District, Shanghai, 200040 China

**Keywords:** Estrogen receptor β, Breast cancer, Biomarker, Prognosis, Mechanism

## Abstract

Breast cancer, a malignant tumor originating from mammary epithelial tissue, is the most common cancer among women worldwide. Challenges facing the diagnosis and treatment of breast cancer necessitate the search for new mechanisms and drugs to improve outcomes. Estrogen receptor (ER) is considered to be important for determining the diagnosis and treatment strategy. The discovery of the second estrogen receptor, ERβ, provides an opportunity to understand estrogen action. The emergence of ERβ can be traced back to 1996. Over the past 20 years, an increasing body of evidence has implicated the vital effect of ERβ in breast cancer. Although there is controversy among scholars, ERβ is generally thought to have antiproliferative effects in disease progression. This review summarizes available evidence regarding the involvement of ERβ in the clinical treatment and prognosis of breast cancer and describes signaling pathways associated with ERβ. We hope to highlight the potential of ERβ as a therapeutic target.

## Background

Breast cancer, a malignant tumor originating from mammary epithelial tissue, is the most common cancer among women worldwide [1]. With the discovery of multiple receptors, the treatment of breast cancer has been greatly advanced. Among them, estrogen receptor (ER) is considered to be important for determining the diagnosis and treatment strategy. In the 1960s, ERα, the first estrogen receptor, was described [2]. After a long period of research, the function of ERα is now well characterized [3], and ERα is widely used for determining medication and imaging strategies [4, 5].

The emergence of the second ER, ERβ, can be traced back to 1996 [6]. After its discovery, much effort has been devoted to the question of the unique functions of ERβ and its potential as a novel target for pharmacological intervention [7, 8]. At present, the widespread expression of ERβ is detected not only in luminal and myoepithelial cells in the normal breast but also in subcutaneous adipose tissue [9] and prostate, testis, uterus, ovary, and brain tissues [10]. Alternations in estrogenic signaling pathways, as well as ERβ expression, have been discussed in the context of physiological and pathological processes, such as maintenance of the bone marrow microenvironment [11], neuronal-mediated contractions of the gastrointestinal tract [12], recovery of reproductive system injury [13], anxiolytic effects [14], and diseases such as Parkinson’s disease [15], endometriosis [16], myocardial infarction [17] and type 2 diabetes [18]. Moreover, ERβ has been shown to participate in the pathological process of various cancers, including uterine leiomyomas [19], colorectal cancer [20], desmoid tumors [21], prostate cancer [22] and duct carcinoma [23].

The association between the activation of ERβ and early transcription and mRNA splicing in breast cancer [24, 25] is well studied. The mechanism of preferential ERβ ligands has also been discussed [26]. However, the significance of ERβ expression and its potential role in normal mammary development and breast cancer remain controversial. In this review, the current state of research addressing the roles of ERβ in breast cancer is summarized, with a particular focus on the past ten years.

### ERβ

Human ERβ is a member of the nuclear transcription factor superfamily, is encoded by the *ESR2* gene (14q23.2), and is composed of 530 amino acids [27]. The DNA binding domain and ligand binding domain of the ERβ protein are 96 and 60% homologous with those of ERα, indicating that they may have similar but not identical functions [28]. ERβ is abundant in the majority of normal breast epithelial cells and is thought to be present in 20–30% of breast cancers [29]. However, as research progresses, the positive rate of ERβ in breast cancer has been reported to be over 60% [30, 31]. The five full-length ERβ variants, which result from alternative splicing of the last coding exon, deletion of coding exons, or alternative usage of untranslated exons and are named ERβ1–5, can be detected in various normal tissues, breast cancer tissues and breast cancer cell lines [32, 33]. Additionally, ERβcx, the carboxy terminal splicing variant of ERβ, plays a role that ERβ cannot [34]. Different isoforms, as well as polymorphisms, may have different associations with tumor characteristics and prognosis [35]. For instance, a study including 150 Iranian women with breast cancer and 147 healthy individuals found that the ERβ polymorphism in exon 7 codon 392 (C1176G) was associated with the occurrence of lymph node metastasis [36]. Genotype frequencies of SNPs, such as rs3020449, rs3020450, rs2987983, rs1271572 and rs1887994 SNP rs4986938, have been discussed [37, 38], and some were found to be associated with increased or decreased risk for breast cancer [38, 39].

### Prognostic value of ERβ in breast cancer

The research regarding the detection of ERβ-like protein established a platform for investigating the relationships between protein expression and patient outcome. The review in 2012 showed that most but not all of the studies indicated an association between higher levels of ERβ-like protein and better clinical outcome, often in patients who were treated with tamoxifen (TAM) [40]. Notably, more than one study found that the expression of ERβ protein had no significant correlation with clinical variables, including tumor size, age, or axillary nodal status [41–43]. Interestingly, a significant decrease in ERβ gene expression in tissues was observed in a cohort of 120 patients with phase II to phase IIIA breast cancer after chemotherapy [44]. However, in another study including 78 women who were postmenopausal with primary stage II to III invasive breast cancer, ERβ did not change when comparing samples from before and after endocrine treatment [45]. Since the role of ERβ in breast cancer remains unclear, the latest studies have made efforts to explore the relationship between ERβ and clinical outcome in many aspects, including the predictive value of ERβ expression [46], the ERβ to ERα ratio [45] and the DNA promoter hypermethylation of ERβ [47], especially in patients who have undergone endocrine therapy [48, 49] and chemotherapy [44, 50]. In general, numerous studies have verified that ERβ is an independent prognostic and/or predictive factor in breast cancer, although the conclusion is still controversial. Studies reporting a correlation of ERβ with clinical outcome for the last 10 years are shown in Table 1.

**Table 1:** ERβ and clinical outcome

ERβ isoform	ERβ expression	ERα status	Number of patients	Clinical outcome	Year (Ref.)
ERβ	↑	–	1400	reduced RFS	2018 [51]
ERβ			32	worse prognosis	2017 [46]
ERβ	↑	+/−	1026	better prognosis	2017 [30]
ERβ	↑		120	worse prognosis	2017 [44]
ERβ		–	17	no association with PFS	2016 [52]
ERβ	↑	+	195	reduced DFS; reduced DFS after endocrine therapy	2016 [49]
Nuclear ERβ1		–	126	no association with DFS and OS	2015 [53]
ERβ	↑	+	127	no association with PFS	2015 [54]
ERβ	↑	–	107	reduced DFS	2015 [54]
Nuclear ERβ1	↑	–	19	reduced OS	2015 [55]
ERβ1		–	571	prolonged OS, DFS, and DMFS	2015 [56]
ERβ	↑	+/−	583	worse prognosis; worse endocrine therapy response	2014 [48]
ERβ	ERα/ERβ: 1–1.5		78	better hormonal treatment response	2013 [45]
ERβ	↓		89	reduced OS	2012 [47]
Nuclear ERβ1	↑	+/−	123	better chemotherapy therapy and endocrine therapy response	2011 [50]
Cytoplasmic ERβ 2/cx		+/−	123	poor chemotherapy response	2011 [50]
ERβ	↓		41	prolonged PFS; better aromatase inhibitor therapy response	2010 [57]
Nuclear P-S105-ERβ	↑	+/−	459	better prognosis	2010 [58]

*RFS* Recurrence-free survival, *PFS* Progression-free survival, *DFS* Disease-free survival, *OS* Overall survival, *DMFS* Distant metastases-free survival.

Multiple studies have now been published in which ERβ shows its anticancer effect and helps predict treatment responsiveness, irrespective of the ERα status. In a prospective cohort of 1026 patients diagnosed with primary invasive breast cancer, ERβ1 positivity, defined as > 75% staining, was associated with a lower risk of breast cancer compared with ERβ1 < 75% tumors. Among 232 patients who underwent chemotherapy, ERβ1 staining > 75% in tumors was associated with a lower risk of breast cancer events. High ERβ1 expression was a favorable prognostic marker in breast cancer, especially in patients receiving chemotherapy [30]. Similarly, another study indicated that positive nuclear ERβ1 expression was correlated with longer 15-year survival in patients treated with TAM [50]. Using mass spectrometry, studies identified serine 105 (S105) in the N-terminus of ERβ, and S105-ERβ immunoreactivity was detected with a higher prevalence and intensity than that of ERβ1 [59]. Nuclear S105-ERβ was associated with better survival, even in tamoxifen-resistant cases [58].

However, there are limited studies that reported opposite results, in which elevated ERβ and cytoplasmic ERβ2 expression predict poor prognosis [50, 57]. For example, Guo et al. showed that compared to patients with low ERβ expression, patients with high ERβ expression in breast cancer tissue displayed a significantly lower median tumor-free survival time [48]. Another study of 195 postmenopausal females with stage I or II ERα-positive breast cancer who underwent endocrine therapy showed that ERβ overexpression results in reduced disease-free survival (DFS) and poor prognosis [49].

Triple-negative breast cancers (TNBCs) lack ERα, progesterone receptor (PR), and human epidermal growth factor receptor-2 (*HER2*) amplification, and due to the poor response to chemotherapy, TNBC patients would benefit greatly if new targeted therapeutics were identified [52]. Scholars have made efforts to find the expression pattern of ERβ in TNBC and determine its significance in prognosis. Evidence has shown that the ERβ expression rate was significantly decreased in patients with TNBC compared with those with triple-positive breast cancer [54]. The conclusions are inconsistent. A study showed that no strong association was found between ERβ1 expression and DFS and overall survival (OS) in a TNBC cohort [53]. However, in a cohort of 571 TNBC patients, ERβ1 predicted better OS, DFS, and distant metastasis-free survival (DMFS) [56]. In contrast, ERβ1 was found to be associated with significantly worse 5-year OS in TNBC patients [55]. Moreover, a study indicated that patients with TNBC and positive ERβ expression exhibited poorer DFS [54] and decreased recurrence-free survival (RFS) regardless of chemotherapy use [51].

### Roles of ERβ in breast cancer therapy

Questions regarding the relationship between the role of ERβ and the treatment of breast cancer have prompted studies about ERβ and drugs. TAM is a standard selective estrogen receptor modulator (SERM) that can be used as an adjuvant therapy for breast cancer recurrence in patients whose primary tumors are ERα positive. The role of ERβ in TAM therapy has been studied [60]. In TAM-treated cells, ERβ overexpression led to an increase in autophagy, which reduced cell viability [61]. ERβ also increased TAM-induced cell death and induced the expression of the proapoptotic gene *BIK* in cooperation with TAM [62]. TAM engaged mitochondrial ERβ as an antagonist, increasing reactive oxygen species (ROS) concentrations from the mitochondria that were required for cytotoxicity [63]. By recruiting ERβ, cJun, cFos, binding protein (CBP), and RNA polymerase II to and dismissing NCoR from the nuclear respiratory factor 1 (NRF-1) promoter, TAM increased NRF-1 expression. Despite this, TAM-induced NRF-1 transcription was likely mediated by ERβ [64]. Additionally, ERβ enhanced the sensitivity of breast cancer cells to TAM [65–67]. ERβ re-expression was thought to sensitize MCF-7/TAM-R cells to the growth inhibitory and proapoptotic effects of TAM, thereby indicating that ERβ re-expression was directly linked to restoring TAM sensitivity [68].

Many drug treatments have been shown to be mediated by ERβ and its isoforms [69–71]. ERβ can enhance the antiproliferative effects of raloxifene [72, 73] and the sensitivity to anti-androgens in TNBC [74]. After treating MCF-7 cells with cisplatin, the overexpression of ERβ contributed to the lower rates of apoptosis, autophagy and ROS production, leading to increased cell survival. The opposite results were found by silencing ERβ in T47D cells [61].

### Mechanisms of ERβ suppressing breast cancer progression

Over the years, researchers have discovered many mechanisms of ERβ in inhibiting tumor progression, especially in breast cancer. ERβ signaling is known to be complex and multifaceted and not just a component of a linear signaling pathway. Overall, the mechanisms associated with ERβ in vivo and in vitro models indicate that ERβ may act as a tumor suppressor.

### The interaction of ERα and ERβ in breast cancer

Some researchers believe that the changes in ERα and ERβ expression in the normal breast support a direct correlation between the nuclear expression of ERs and the proliferative nature of the breast [75]. The relative levels of ERβ and ERα in breast cancer are related to the activities of multiple signaling pathways responsible for cell proliferation and endocrine therapy response [45, 76]. A study shows that under the condition of coexpression of ERα and ERβ, HER2 expression is frequently found to be negative, whereas the Ki-67 index is upregulated, indicating an association between this special combination of biomarkers and breast cancer aggressiveness [46]. Furthermore, elevated ERβ can affect ERα expression at the transcriptional level through downregulation of basal ERα promoter activity. The proximal GC-rich motifs at − 223 and − 214 are essential for ERβ-induced ERα downregulation in breast cancer cells. This downregulation of ERα occurs through ERβ-Sp1 protein-protein interactions within the ERα promoter region and recruitment of a corepressor complex containing the nuclear receptor corepressor NCoR, hypoacetylation of histone H4 and displacement of RNA-polymerase II [77]. The use of an ERβ-specific agonist significantly decreases the expression and functional activity of ERα in MCF-7 breast cancer cells, accompanied by decreased transcription of a downstream effector, breast cancer-associated gene 2 (*BCA2*) [78].

Additional evidence shows that tumors with a low ERα/ERβ ratio have increased oxidative damage, antioxidant enzyme protein levels and uncoupling protein (UCP) and sirtuin 3 (SIRT3) protein levels. Glutathione peroxidase, complex V, complex III, complex II, complex IV, protein kinase B (AKT), stress-activated protein kinase (SAPK), and ERα are positively correlated with the ERα/ERβ ratio, while carbonyl groups, catalase, CuZn-superoxide dismutase, UCP5, SIRT3, and ERβ are negatively correlated with the ERα/ERβ ratio [79]. It is reasonable to suggest that the imbalance of two estrogen receptors may lead to the occurrence of breast cancer .

### Phosphatidylinositol-3-kinase (PI3K)/AKT pathway

**Fig. 1:**
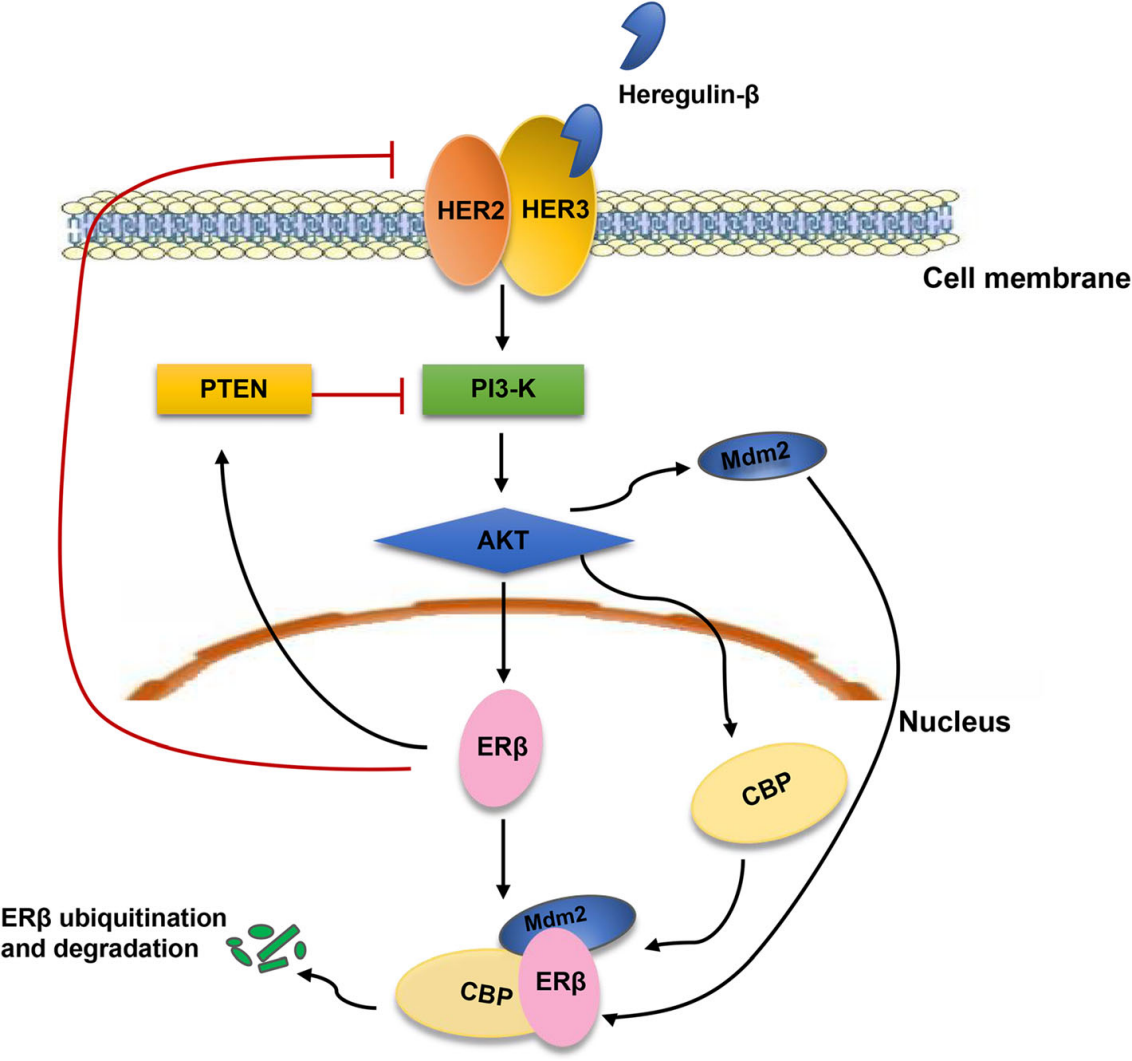
The interaction between ERβ and PI3-K/Akt signaling. Heregulin**-**β binds to HER2/HER3 receptors, which leading to the PI3-K/Akt signaling activation. The enhancement of PI3-K/Akt signaling triggers the recruitment of the E3 ubiquitin ligase *MDM2* to ERβ, which is further stabilized by CBP, resulting in ERβ polyubiquitination. On the contrary, expression of ERβ results in a decrease in Akt signaling by increasing *PTEN* levels and decreasing protooncogene HER2/HER3 signaling

The PI3K/AKT pathway is a common pathway in tumors and is negatively regulated by phosphatase and tensin homolog deleted on chromosome ten (*PTEN*). This pathway can regulate cellular proliferation, invasion, apoptosis, and hypoxia-related protein upregulation [80]. Indeed, the activation of the PI3K/AKT pathway acts as an important mechanism of ERβ downregulation in breast cancer and is thought to be associated with *PTEN* [56, 81]. The enhancement of PI3-K/Akt signaling and synergistic activation of coactivator cAMP response element-binding (CREB)-CBP induces ERβ ubiquitination and degradation. This process is potentiated by a negatively charged hinge region of ERβ. Activated Akt triggers the recruitment of the E3 ubiquitin ligase *MDM2* to ERβ, which is further stabilized by CBP, resulting in ERβ polyubiquitination [82]. By increasing *PTEN* levels and decreasing protooncogene HER2/HER3 signaling, Akt signaling is reduced. Expression of ERβ in ERα-positive T47D and MCF-7 cells results in a decrease in Akt signaling and a decrease in the active form of an upstream regulator of Akt, the HER2/HER3 receptor dimer [65] (Fig. 1).

### Roles of ERβ in breast cancer cell migration and invasion

It must be pointed out that ERβ plays an important role in the cell behavior and extracellular matrix (ECM) composition of breast cancer cells and may have an effect on important chemokine receptors [83, 84]. It is also involved in the beclin1-dependent autophagic cascade [85].

To understand the underlying mechanisms of ERβ in the migration of breast cancer, Piperigkou Z et al. knocked down the human ERβ gene to suppress ERβ expression in MDA-MB-231 breast cancer cells. The downregulation of ERβ decreases the expression of fibronectin and vimentin while increasing the expression of E-cadherin and cell junctions. In addition, ERβ plays a vital role in the gene expression of proteoglycans syndecans-2/− 4 and serglycin, several matrix metalloproteinases, plasminogen activation system components and receptor tyrosine kinases. The downregulation of ERβ prevents breast cancer cell migration through tyrosine kinase receptor [epidermal growth factor receptor (EGFR)/insulin-like growth factor-I receptor (IGF-IR)] and Janus kinase/signal transducer and activator of transcription (JAK/STAT) signaling pathways [86].

However, ERβ1 is found to have an opposite regulatory effect on E-cadherin. Considering the positive correlation between the expression of ERβ1 and E-cadherin in breast cancer samples, Thomas and colleagues investigated the role of ERβ1 in epithelial-mesenchymal transition (EMT) and basal-like breast cancer cell invasion. In a subsequent work, they concluded that ERβ1 inhibits EMT and invasion in basal-like breast cancer cells when growing either in vitro or in vivo in zebrafish. The inhibition of EMT is related to the upregulation of miR-200a/b/429 by ERβ1 and the subsequent repression of zinc finger E-box-binding homeobox 1 (ZEB1) and smad interacting protein (SIP1), which contributes to the increased expression of E-cadherin. By stabilization of the ubiquitin ligase c-Cbl complexes and subsequent ubiquitylation and degradation of the activated receptor, EGFR, a basal marker, is downregulated. This process is involved in ERβ1-mediated EMT inhibition and EGFR signal transduction, which eliminates the ability of ERβ1 to sustain the epithelial phenotype [87]. A similar function of ERβ1, in which it downregulates ZEB1 and thereby regulates the expression of E-cadherin, is also found in AR-positive TNBC cells. The activated AR in TNBC cells upregulates the transcription of ERβ1, which subsequently suppresses ZEB1 [88].

**Fig. 2:**
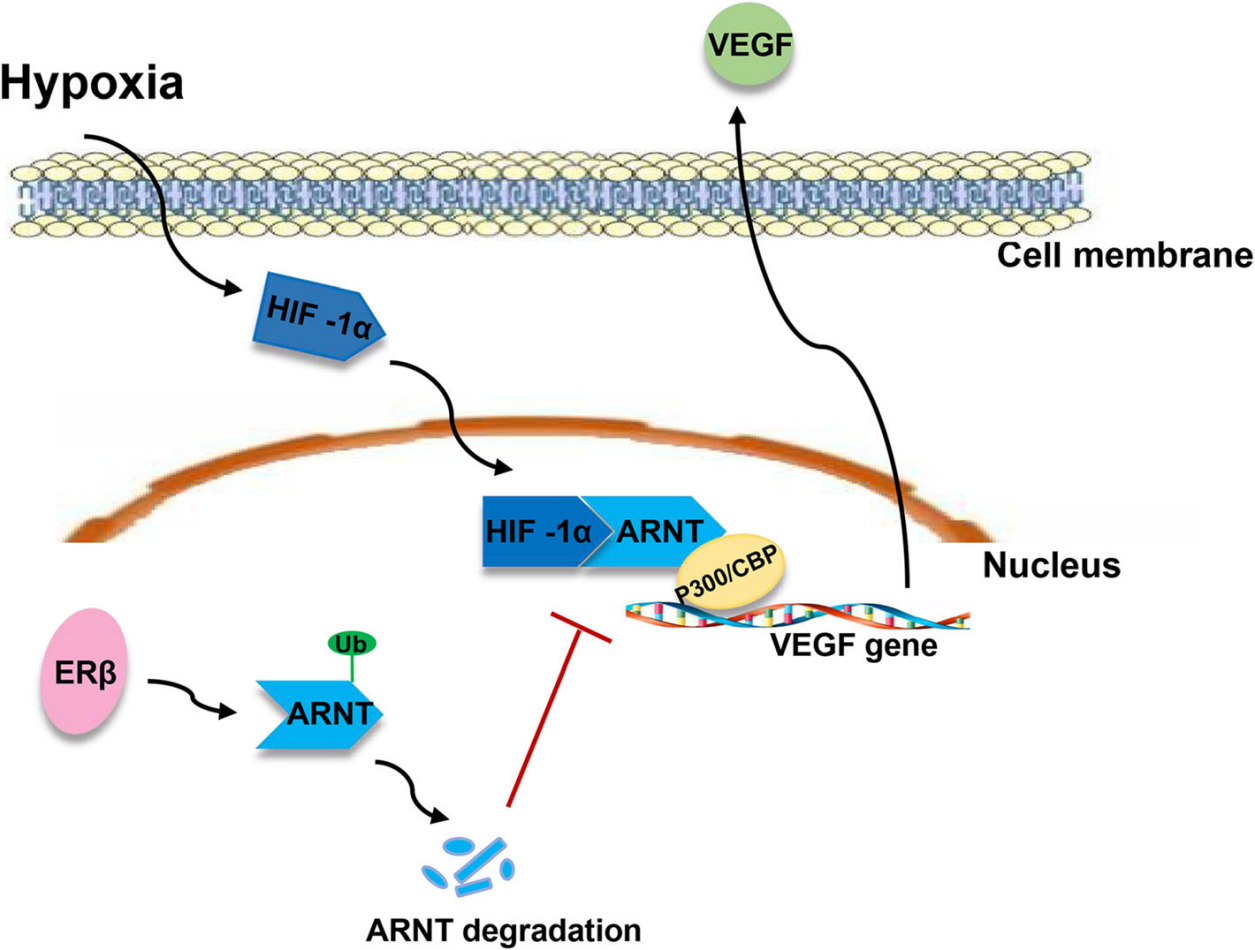
ERβ reduces VEGF transcription via reducing HIF-1α/ARNT complexes. ERβ attenuated the hypoxic induction of VEGF mRNA by directly reducing HIF-1α/ARNT complexes binding to VEGF gene promoter. The inhibition of HIF-1α activity is related to the ability of ERβ to degrade ARNT via ubiquitination processes, leading to the reduction of active HIF-1α/ARNT complexes

ERβ elicites tumor-suppressive effects, particularly with regard to suppression of metastatic phenotypes, which is characterized by the induction of a family of secreted proteins known as cystatins and the subsequent inhibition of canonical transforming growth factor (TGF-β) signaling [89], leading to decreased expression of a network of genes related to extracellular matrix, cell invasion and vitamin D3 metabolism [90]. There is evidence that ERβ is involved in angiogenesis in breast cancer. ERβ decreases the expression of the proangiogenic factors vascular endothelial growth factor (VEGF) and platelet-derived growth factor β (PDGFβ) in T47D breast cancer cells and reduces the number of intertumoral blood vessels. The expression of ERβ in cell culture results in decreased VEGF expression and PDGFβ mRNA under normoxic as well as hypoxic conditions and reduces secreted VEGF and PDGFβ protein levels in cell culture medium [91]. Furthermore, ERβ attenuates the hypoxic induction of VEGF mRNA by directly reducing hypoxia inducible factor (HIF-1α) binding to the VEGF gene promoter [92]. The inhibition of HIF-1α activity by ERβ expression is related to the ability of ERβ to degrade aryl hydrocarbon receptor nuclear translocator (ARNT) via ubiquitination processes, leading to the reduction of active HIF-1α/ARNT complexes [93]. These results show that ERβ is capable of inhibiting HIF-1α-mediated transcription by downregulating ARNT, which can participate in the tumor suppressive function of ERβ (Fig.2).

Other experimental studies have demonstrated that ERβ overexpression can also increase the level of integrin αI [94], growth-inhibitory p21/WAF and epithelial cell marker cytokeratin 8 [66], consequently modulating adhesion and migration of breast cancer cells.

### The inhibitory effect of ERβ in cell cycle progression

Several lines of evidence suggest that the inhibitory effect of ERβ is due to blockade of cell cycle progression. ERβ knockdown results in a significant growth of several breast cancer cells, accompanied by elevated cyclin A2 expression [66] and mitofusin 2 (mfn2) [95].

After being treated with a combination of ERβ agonist and letrozole, tumors from mice presents an increase in ERβ levels compared to those with single drug treatment. Subsequently, decreased cyclin D1 and increased cyclin D1/CDK inhibitors p21 and p27 levels are observed, suggesting that the combination therapy may inhibit tumor growth by blocking G1/S phase cell cycle progression [96]. In TNBC, ERβ expression inhibits cell growth by inducing G1 cell cycle arrest, which was further enhanced by 17β-estradiol treatment [97].

ERβ is also involved in the G2 cell cycle [98]. Gene expression studies and ingenuity pathway analysis have identified a network of ERβ-regulated genes related to cell cycle progression. ERβ causes G2 cell cycle arrest by repressing c-myc, cyclin D1, and cyclin A gene transcription and increasing the expression of p21 (Cip1) and p27 (Kip1), which leads to inhibition of proliferation [99].

Unliganded ERβ inactivates CDK1 by inhibiting cyclin B1 and stimulating the expression of GADD45A and BTG2, which eventually results in G2 cell cycle arrest [100, 101]. The activation of ERβ results in decreased proliferation rates and stagnation of the cell cycle, thus inhibiting the growth of tumors.

### The role of ERβ in regulating mitochondrial function

ERβ can perform its role in tumor inhibition by regulating mitochondrial function and dynamics. After overexpression of ERβ in the T47D-ERβ tetracycline-inducible cell line was inhibited, a decrease in mitochondrial biogenesis is observed, together with fewer fission events [102]. The trifunctional protein beta subunit (HADHB), a mitochondrial protein that is required for β-oxidation of fatty acids, colocalized with ERβ. Silencing of ERβ results in enhanced HADHB enzyme activity. This suggests that ERβ causes a significant reduction in HADHB enzyme activity and thus affects cellular oxidative stress through influencing the rate of β-oxidation of fatty acids in breast cancer cells [103].

### The interaction among ERβ and other proteins

Many substances have been found to interact with ERβ. Koyama et al. reported that deleted in breast cancer 1 (*DCB1*) can interact with ERβ by a direct interaction between the amino-terminus of *DCB1* and the activation function-1/2 domain of ERβ, which is similar to ERα. This interaction inhibits the transcriptional activation of ERβ, as well as the transcriptional activation of ERβ on the downstream apoptosis-related gene *BCL-2* [104].

A new ERβ1-interacting protein, inhibitor of differentiation-1 (Id1), is identified by a yeast two-hybrid screening technique; this protein interacts with ERβ1 via the helix-loop-helix domain of the Id1 protein. Id1 is the dominant negative regulator of basic helix-loop-helix (bHLH) transcription factors, which promote cell proliferation in breast cancer cells. ERβ binds with Id1 protein, whilst removing Id1 inactivation of p21 expression, resulting in decreased cancer cell growth [105].

ERβ is a new target of small ubiquitin-related modifier (SUMO-1). Due to further regulation by phosphorylation of additional adjacent serine residues by glycogen synthase kinase 3β (GSK3β), ERβ SUMOylation is maximized in response to hormones. SUMO-1 prevents ERβ degradation by competing with ubiquitin at the same receptor site and inhibits ERβ transcription by changing estrogen-responsive target promoter occupancy and gene expression in breast cancer cells [106].

### DNA methylation, microRNAs (miRNAs) and oncogenes

Evidence has confirmed that ER β exerts its oncosuppressive role via regulation of gene transcription and RNA maturation and posttranscriptional regulation of RNA activity [25, 107, 108], providing novel information on the biological role of ERβ in breast cancer.

### DNA methylation

Many studies have confirmed that ERβ gene silencing mediated by DNA methylation is an important mechanism in breast cancer [109]. Compared to those of adjacent normal tissues, the methylation level of breast cancer is significantly higher, which may be the reason for the decreased ERβ gene expression [110]. According to Mirza et al., patients with concurrent hypermethylation of ERβ and retinoic acid receptor β2 (RARβ2) showed a significantly shorter median OS [47]. Interestingly, another study found that the methylation level of the ON promoter could be a more reliable parameter for prognosis in breast cancer than ERβ1 mRNA and/or protein levels [111]. Therefore, these findings may indicate the role of *ERS2* methylation in breast cancer development and treatment.

### MicroRNAs (miRNAs) miRNAs

miRNAs are small noncoding RNAs that participate in the regulation of gene activity and tumorigenesis [112]. Evidence has demonstrated that miRNAs are involved in regulating the expression of ERβ. By targeting its 3′-UTR, miR-92 downregulates ERβ1 expression. Inhibition of miR-92 in MCF-7 cells induces ERβ1 expression in a dose-dependent manner. This may be an important mechanism for the downregulation of ERβ expression in breast cancer [113]. ERβ expression also causes changes in miRNA expression. The comparison of miRNAome expression in ERβ + and ERβ- hormone-responsive breast cancer reveals 67 miRNAs with distinct different expression patterns [114]. The upregulation of some miRNAs exerts an antiproliferative effect in breast cancer [115]. Examples of miRNAs regulated by ERβ are listed in Table 2.

**Table 2:** Examples of miRNAs regulated by ERβ

	miRNA	Mechanism	Year (Ref.)
Upregulated	miR-145	inhibition of EMT	2017 [116]
	miR-30a-5p	inhibition of EMT	2014 [117]
	miR-200a/b/429	inhibition of EMT and invasion	2012 [87]
Downregulated	miR-181a-5p	inhibition of cholesterol biosynthesis	2020 [118]
	miR-10	regulation of ECM composition	2017 [116]
	miR-375	suppression of proliferation	2013 [115]

*EMT* Epithelial-mesenchymal transition, *ECM* Extracellular matrix

ERβ upregulates miR-200a/b/429 to inhibit EMT and invasion in basal-like breast cancer cells both in vitro and in vivo. The upregulation of miR-200a/b/429 leads to a decrease in downstream ZEB1 and SIP1, which triggers increased E-cadherin expression. Additionally, ERβ1 is found to be correlated with E-cadherin expression in breast cancer samples [87]. Additionally, ERβ-induced downregulation of miR-10b and upregulation of miR-145 [116] and miR-30a-5p [117] are found to influence the extracellular matrix (ECM) composition and significantly reduce the aggressiveness of breast cancer cells.

In ERβ + TNBC, ERβ induces miR-181a-5p overexpression, which is involved in the inhibition of the cholesterol biosynthesis pathway in TNBC cells [118]. These results suggest that miRNA regulation might be a critical event in the control of the biological and clinical phenotype of breast cancer by ERβ.

#### P53

ERβ is an activator of wild-type *P53*-dependent transcription and is thought to interact with *P53*. The upregulation of ERβ or activation with ERβ agonists results in increased nuclear *P53* expression [119, 120].

The synergistic effect of ERβ and *P53* inactivation functions is an important aspect of the occurrence and development of breast cancer [121]. In TNBC, *P53* status, together with the *ESR2* mutant, show antiproliferative effects [122]. ERβ1 upregulates target genes of mutant *P53* that are associated with a normal phenotype and downregulated prometastatic factors [123]. Somatic loss of ERβ and *P53* accelerates tumor development in a mouse model of mammary tumors [121]. ERβ expression leads to abrogation of S-phase and Chk1/Cdc25C-mediated G_2_/M checkpoints after cisplatin and doxorubicin exposure. Interestingly, this effect is found only in *P53*-defective breast cancer cells but not in *P53* wild-type mammary cells [124].

Moreover, ERβ’s antiproliferative and proapoptosis effects in breast cancer cells involve the interaction of *P53* and ERα. ERβ reduces ERα-*P53* binding by interacting with *P53*, resulting in antagonization of ERα-*P53*-mediated transcriptional regulation. Additionally, ERβ stimulates the accumulation of histone H3 lys4 trimethylation (H3K4me3) and RNA polymerase II on ERα-repressed genes, which leads to the epigenetic activation of H3K4me3-related suppressor gene transcription, thus promoting *P53*-based tumor suppression. ERβ also attenuates the crosstalk between ERα and *P53* by reducing corepressor NCoR and SMRT recruitment by ERα [125].

#### Breast cancer 1 (BRCA1)/ breast cancer 2 (BRCA2)

Hereditary breast cancer can account for 5 to 10% of all breast cancer patients [126], and *BRCA1/BRCA2* mutations can be detected in more than 60% of hereditary breast cancer patients [127]. In *BRCA1*-associated hereditary breast cancer, the expression of ERβ is significantly higher than that of ERα [128] . Therefore, the effect of estrogen in these breast cancer patients may be mainly mediated by ERβ [129]. In addition, *BRCA1* mutations are common in TNBC [130], and there have been many pharmacological and nutritional studies on the relationship between BRCA1 and ERβ. Studies have shown that large amounts of soy food can reduce the risk of breast cancer and improve the prognosis of breast cancer patients [131, 132] . This is mainly related to genistein, which is one of the main components of soy isoflavones and can inhibit several steps of carcinogenicity [133] . Genistein can trigger transactivation with ERβ from estrogen response element-reporter genes and is 20- to 40-fold more potent in stimulating ERβ-mediated transcription in MCF-7 cells [134] . Besides, genistein strongly inhibits the growth of *BRCA1* mutant cells, but only has a weaker effect in cells expressing wild-type *BRCA1* protein. The hypersensitivity of this genistein to *BRCA1* mutant cells may be related to the higher expression of ERβ [135]. The relationship between ERβ and *BRCA1* in breast cancer still needs further basic research to verify. As for the relationship between *BRCA2* and ERβ, there still a lack of research.

## Conclusion and outlook

Over time, more functions and activation mechanisms mediated by ERβ have been discovered. An increasing body of evidence suggests that ERβ is thought to be a protective factor that suppresses uncontrolled proliferation, which is mediated by concentration-dependent and cell line-specific effects on cell growth and gene expression [136]. In addition, ERβ can exert its antitumor effect via gene transcription and miRNA regulation. In the clinical aspect, although the conclusions are controversial, ERβ can predict clinical outcome and response to chemotherapy or endocrine therapy. Here, we have discussed some new progress regarding the role of ERβ, which merits further investigation.

Clearly, continued efforts are needed to understand the nature and function of ERβ, which may offer clinical evidence when the diagnosis of breast cancer patients is ambiguous and provide a new prospect for the management of breast cancer.

## Data Availability

Not applicable.

## Abbreviations

ER: Estrogen receptor
TAM: Tamoxifen
RFS: Recurrence-free survival
PFS: Progression-free survival
DFS: Disease-free survival
OS: Overall survival
DMFS: Distant metastases-free survival
TNBC: Triple-negative breast cancer
PR: Progesterone receptor
HER2: Human epidermal growth factor receptor-2
SERM: Selective estrogen receptor modulator
ROS: Reactive oxygen species
CBP: Binding protein
NRF-1: Nuclear respiratory factor 1
BCA2: Breast cancer associated gene 2
UCP: Uncoupling protein
SIRT3: Sirtuin 3
AKT: Protein kinase B
SAPK: Stress-activated protein kinase
PI3-K: Phosphatidylinositol-3-kinase
PTEN: Phosphatase and tensin homolog deleted on chromosome ten
CREB: cAMP response element-binding
ECM: Extracellular matrix
EGFR: Epidermal growth factor receptor
IGF-IR: Insulin-like growth factor-I receptor
JAK/STAT: Janus kinase/signal transducer and activator of transcription
EMT: Epithelial-mesenchymal transition
ZEB1: Zinc finger E-box-binding homeobox 1
SIP1: Smad interacting protein
TGF-β: Transforming growth factor
VEGF: Vascular endothelial growth factor
PDGF β: Platelet-derived growth factor β
HIF -1α: Hypoxia inducible factor-1α
ARNT: Aryl hydrocarbon receptor nuclear translocator
mfn2: Mitofusin2
RARβ2: Retinoic acid receptor β2
HADHB: Trifunctional protein, beta subunit
DCB1: Deleted in breast cancer 1
Id1: Inhibitor of differentiation-1
bHLH: Basic helix-loop-helix
SUMO-1: Small ubiquitin-related modifier
GSK3β: Glycogen synthase kinase 3β
MicroRNAs: miRNAs
H3K4me3: Histone H3 lys4 trimethylation
BRCA1: Breast cancer 1
BRCA2: Breast cancer 2
